# Axial length as a predictor of myopic macular degeneration: a meta-analysis and clinical study

**DOI:** 10.1038/s41433-025-03782-6

**Published:** 2025-04-25

**Authors:** Tong Mu, Hai-Long He, Xuan-Yu Chen, Yu-Xin Fang, Jie Xu, Zi-Bing Jin

**Affiliations:** Beijing Institute of Ophthalmology, Beijing Tongren Eye Center, Beijing Tongren Hospital, Capital Medical University, Beijing Ophthalmology & Visual Science Key Laboratory, Beijing, China

**Keywords:** Predictive markers, Predictive markers

## Abstract

**Purpose:**

This study aims to investigate the relationship between axial length (AL) and the severity of myopic macular degeneration (MMD).

**Methods:**

We conducted a comprehensive search of PubMed, Web of Science, and China National Knowledge Infrastructure (CNKI) databases from their inception until October 1, 2023, to identify population-based or hospital-based studies reporting AL across different grades of MMD. Only studies employing the International Photographic Classification and Grading System for Myopic Maculopathy (META-PM) were included. A meta-analysis was performed to assess the association between AL and MMD severity. To further validate our findings, we analyzed data from 395 eyes of 206 participants at Beijing Tongren Hospital, Capital Medical University.

**Results:**

The meta-analysis included 20 high-quality studies from seven countries, with 33822 patients studied. Information, including the study name, year of publication, country, sample size, basic demographic characteristics of the participants, AL of different grades of MMD, best corrected visual acuity (BCVA), and spherical equivalent (SE), was extracted. The meta-analysis revealed a significant overall increase in AL as MMD progressed from category C0 to C4 (*P* < 0.0001). AL exhibited a consistent increasing trend from categories C0 to C3; however, this trend appeared to level off between categories C3 and C4, with no further increase observed. This trend was confirmed by the distribution of our new dataset. A higher prevalence of MMD was significantly associated with longer AL (per 1 mm increase: OR, 1.90; 95% CI, 1.75–2.07; *P* < 0.001), older age (per 1-year increase: OR, 1.04; 95% CI, 1.02–1.05; *P* < 0.001), and female gender (OR, 1.89; 95% CI, 1.24–2.89; *P* < 0.01). Compared with C0, each 1 mm increase in AL was associated with an increasing likelihood of MMD progression, with ORs of 2.8 for C1, 3.6 for C2, 5.2 for C3, and 5.7 for C4. The increase in OR was more pronounced in later stages (C2–C3 and C3–C4) than in earlier transitions (C0–C1 and C1–C2). Similarly, the ORs for age increased significantly from C3 to C4, and the ORs for female gender increased progressively from C2 to C4.

**Conclusions:**

The meta-analysis and new clinical study indicate a clear trend of increasing AL with advancing MMD severity from C0 to C4. However, the relationship between AL and MMD progression from C3 to C4 warrants further investigation. Additionally, older age and female gender are identified as risk factors for MMD progression.

## Introduction

Myopia has become a public health issue worldwide for its increasingly high prevalence [1–3]. It is estimated that by 2050, 49.8% of the world’s population will be myopic, with 9.8% being highly myopic [4]. Pathologic myopia (PM), characterized by degenerative changes in the choroid, retina, and sclera, is a leading cause of irreversible visual impairment in East Asia [5–12]. PM can cause various sight-threatening complications [1, 13, 14], such as myopic macular degeneration (MMD), posterior staphyloma, myopic optic neuropathy, and retinal detachment. MMD is particularly concerning due to its irreversible nature and severe visual consequences, underscoring the need for early diagnosis and intervention.

Axial length (AL) is a key biometric parameter that reflects the combined measurements of anterior chamber depth, lens thickness, and vitreous chamber depth in the eye. It is not only a major factor in the development of myopia and high myopia but also a strong risk factor for MMD. AL measurement is generally performed using optical biometry devices, including those based on partial coherence interferometry and more recent systems incorporating optical coherence tomography (OCT), which are non-contact methods that are easy to operate. While the role of AL in MMD is well-established, the exact nature of its relationship with MMD severity remains unclear. According to the International Photographic Classification and Grading System for Myopic Maculopathy (META-PM) [15], MMD was categorized into five categories: “no myopic retinal degenerative lesion”, “tessellated fundus”, “diffuse chorioretinal atrophy”, “patchy chorioretinal atrophy”, and “macular atrophy”, respectively from Category 0 (C0) to Category 4 (C4). Zhao et al. reported a significant increase in AL from category C0 to C3 (*P* < 0.01), but no difference was observed between C3 and C4 (*P* > 0.05) [16]. Similarly, Fang et al. also suggested that progression from C3 to C4 is uncommon [3]. These findings raise important questions about whether AL influences MMD severity in the same way across different MMD categories. This study aims to deepen our understanding of the relationship between AL and MMD progression, specifically by determining whether AL exerts the same influence on each MMD category and quantifying its impact. These findings could help refine our understanding of MMD progression and guide future clinical management.

Previous meta-analyzes on myopia have predominantly focused on environmental risk factors [17–19], intervention for myopia control [20, 21], and the relationship between myopia and other diseases such as glaucoma and cataract [22, 23]. However, the specific role of AL in the pathogenesis and progression of MMD remains underexplored. Despite AL being a well-established determinant in the progression of myopia, no systematic analysis has directly examined its relationship with the severity of MMD. This study seeks to address this gap by investigating whether AL influences MMD severity uniformly across different MMD categories, particularly exploring whether AL exhibits a distinct pattern between C3 and C4. This hypothesis is based on the clinical observation that the progression from C3 to C4 may not be directly linked to changes in AL, suggesting that AL may stabilize at more advanced stages of the disease. By quantifying the impact of AL on MMD progression, we aim to refine our understanding of AL’s role as a critical risk factor in MMD, with implications for future diagnostic and therapeutic strategies. In order to address this, we first summarized the results of published studies and conducted a meta-analysis to investigate the association between AL and the severity of MMD. Second, we validated our findings with new data collected from the ophthalmic clinic of Beijing Tongren Hospital.

## Materials and methods

### Meta-analysis

This analysis was performed according to the Preferred Reporting Items for Systematic Reviews and Meta-Analyzes (PRISMA) and registered on the PROSPERO website (https://www.crd.york.ac.uk/prospero/; ID: CRD42023447051). All the following processes were performed according to the instructions of our registration.

#### Search strategy and selection criteria

We searched 3 databases for literature retrieval: PubMed, Web of Science, and China National Knowledge Infrastructure (CNKI) from inception to October 1, 2023, without any language restrictions. We also carefully read the reference lists of the included studies and previous meta-analyzes to identify other potential studies. The search terms were as follows:

1.1.1 PubMed: (“axial length”[tw] OR “Axial Length, Eye”[Mesh]) AND (“myopic macular degeneration”[tw] OR “myopic maculopathy*“[tw] OR “myopic retinopathy*“[tw] OR “Macular Degeneration”[Mesh])

1.1.2 Web of Science: (TI = “axial length” OR AB = “axial length” OR TS = “Axial Length, Eye”) AND (TI = “myopic macular degeneration” OR AB = “myopic macular degeneration” OR TI = “myopic maculopathy*“ OR AB = “myopic maculopathy*“ OR TI = “myopic retinopathy*“ OR AB = “myopic retinopathy*“ OR TS = “Macular Degeneration”)

1.1.3 CNKI: (TKA = “axial length” OR SU = “Axial Length, Eye”) AND (TKA = “myopic macular degeneration” OR TKA = “myopic maculopathy*“ OR TKA = “myopic retinopathy*“ OR SU = “Macular Degeneration”)

#### Study selection

Two trained reviewers (TM and HLH) independently screened the titles, abstracts, and full texts to identify relevant literature. Any disagreement arising between the two reviewers was resolved by a third reviewer (JX). Our inclusion criteria were as follows: (1) population-based or hospital-based study; (2) detailed description of the definition and classification of MMD; and (3) information on AL for different grades of MMD. The exclusion criteria were as follows: (1) case reports, animal experiments, and reviews; (2) unavailable full text; and (3) classification of MMD other than META-PM.

According to the International Photographic Classification and Grading System for Myopic Maculopathy (META-PM) [15], MMD was defined as myopic macular changes equal to or severer than diffuse chorioretinal atrophy (C2), and/or any “plus” lesions including lacquer cracks, myopic choroidal neovascularization (CNV), and Fuchs spot.

#### Data extraction and quality assessment

Two trained investigators (TM and HLH) extracted data from the included studies. Any disparity between them was resolved by discussion with a third investigator (JX). Information, including the study name, year of publication, country, sample size, basic demographic characteristics of the participants, AL of different grades of MMD, best corrected visual acuity (BCVA), and spherical equivalent (SE), was extracted.

The quality assessment was conducted by two investigators (TM and HLH) independently according to the Newcastle-Ottawa Scale (NOS). Each selected study was evaluated and given a score of 0–9. A score of 7–9 was considered high quality, 4–6 was moderate quality, and less than 4 was considered low quality. Studies considered of low quality were excluded from the analysis.

#### Statistical analysis

R software was used for meta-analysis. The mean axial length (AL) was utilized as the outcome variable for the meta-regression, as AL is a continuous variable. For the effect size, we provided the point estimate along with its corresponding 95% confidence intervals (CI). This approach enabled us to appropriately analyze and present the results for these continuous outcome measures. The median, maximum, and minimum data mentioned in the included studies were transformed according to the formula and then combined for analysis. To assess the heterogeneity among the included studies, we conducted a *χ*^2^ test (with a significance level of α = 0.1) and evaluated it using the *I*^2^ statistic. If the heterogeneity test result *I*^2^ > 50%, it indicates that there was statistical heterogeneity among the results of each study. In such cases, we would identify potential causes of significant heterogeneity and assess whether the selection criteria need to be improved and subgroup analyses performed. When this was not feasible, we would implement the removal of studies that contributed significantly to the elevated heterogeneity. A *P* value < 0.05 was considered statistically significant.

### New clinical study

A total of 206 participants who visited Beijing Tongren Hospital, Capital Medical University, and underwent ophthalmological examinations, including AL measurements and color fundus photography of both eyes from May 8, 2024, to August 8, 2024, were included in our study. AL was measured using the IOL Master (700, Zeiss, Germany). Fundus photographs were obtained from both eyes of each participant to contain macular and optic nerve head areas using 45-degree nonmydriatic Topcon (TRC-NW400, Topcon, Japan) or Canon (CR-DGi, Canon, Japan) equipment.

Two experienced ophthalmologists (TM and HLH) classified the fundus photographs based on the META-PM system. When their judgments clashed, the photographs were re-examined by a third experienced ophthalmologist (JX). They were blinded to the clinical data of participants during the photograph evaluation. This study received approval from the Ethics Committee of Beijing Tongren Hospital, and all procedures adhered to the principles outlined in the Declaration of Helsinki (TREC2022- KY045). Given that this was a retrospective study involving noninvasive methods and the data collected were deidentified, informed consent was waived for study participants.

The data was visualized with a box plot using ChiPlot (https://www.chiplot.online/). Statistical analysis was performed using IBM SPSS Statistics 27.0.1 (IBM Corporation, New York, USA). One-way ANOVA and *t*-test were performed to compare whether there were significant differences in the mean value of AL among different groups of MMD categories. Unless otherwise indicated, data are expressed as mean (Standard Deviation [SD]). Regression analysis was performed to identify the association between AL and MMD categories. Linear mixed-effects model was used to eliminate the effect of including binocular data from the same patient. The odds ratios (ORs) and their 95% CIs for the development and progression of myopic maculopathy were calculated by using ordinal Logistic regression model and multinomial Logistic regression model, which are multivariable models. A *P* value < 0.05 was considered statistically significant.

## Results

### Meta-analysis

#### Selection of studies and quality assessment

**Fig. 1:**
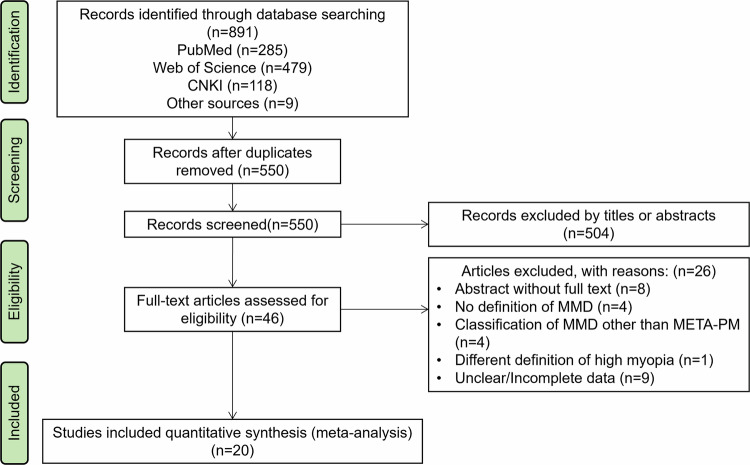
Flow chart of literature selection.

We conducted a systematic search from three databases, with 891 initial records included. After careful selection, 20 studies were identified according to the inclusion and exclusion criteria and were included for qualitative analysis (Fig. 1). According to the Newcastle-Ottawa Scale (NOS), all 20 studies were considered to be of high quality. Detailed information on quality assessment was provided in sTable 1.

#### Study characteristics

The basic characteristics of the included studies were summarized in Table 1. Of the 20 studies [3, 16, 24–41], 17 were conducted in Asia [3, 16, 24,27–38, 40, 41], two in Europe [25, 26], and one in North America [39]. Three studies reported AL data for all categories of MMD (C0-C4) [16, 31, 37], and the other 17 studies reported AL data for only partial categories of MMD.

**Table 1:** Basic characteristics of included studies in the meta-analysis.

Country	Study	Year	Sample size	Age (years)	Sex	AL (mm)	BCVA/VA	SE (D)	Quality assessment
China	Wang et al.	2012	57 eyes 29 patients	C1: 35.5 ± 10.20 C2: 41.21 ± 11.32	/	C1: 27.55 ± 0.94 C2: 29.54 ± 1.63	C1: 0.02 ± 0.03 C2: 0.42 ± 0.42	C1: −11.93 ± 2.24 C2: −15.44 ± 4.23	High
	Yan et al.	2018	2695 patients	2001: 54.6 ± 9.8	/	C1: 26.26 ± 1.61 C2: 27.68 ± 2.26 C3: 29.90 ± 0.59 C4: 29.0	C1: 0.16 ± 0.25 C2: 0.50 ± 0.47 C3: 0.91 ± 0.79 C4: 2.0	C1: −8.48 ± 2.73 C2: −11.42 ± 4.12 C3: −14.65 ± 5.08 C4: −16.25	High
	Zhou et al.	2018	115 eyes 67 patients	C0: 37.2 ± 9.9 C1: 34.3 ± 10.5	/	C0: 26.12 ± 0.74 C1: 27.04 ± 0.71	/	C0: −7.882 ± 1.017 C1: −8.813 ± 1.647	High
	Xiao et al.	2018	884 eyes and patients	C0: 15.8 (13.2, 21.6) C1: 23.5 (17.2, 33.9) C2: 26.4 (16.9, 40.4) C3: 34.0 (27.4, 47.7) C4: 48.3, 54.7	C0: 237 men, 267 women C1: 81 men, 96 women C2: 85 men, 93 women C3: 7 men, 16 women C4: 0 men, 2 women	C0: 26.8 (26.1, 27.5) C1: 27.5 (26.7, 28.4) C2: 29.0 (28.0, 30.3) C3: 30.4 (29.1, 31.3) C4: 31.2, 27.7	/	C0: −8.13 (−9.25, −7.25) C1: −9.25 (−12.19, −8.00) C2: −13.13 (−16.50, −10.25) C3: −18.25 (−20.50, −16.13) C4: −20.88, −9.75	High
	Liu et al.	2020	857 eyes	22.2 ± 12.1	402 men, 455 women	C0,C1: 27.0 ± 1.1 C2: 29.1 ± 1.8	C0,C1: 0.06 ± 0.15 C2: 0.26 ± 0.25	C0,C1: −8.9 ± 2.2 C2: −13.7 ± 4.3	High
	Zhao et al.	2020	1841 eyes 1132 patients	C0: 32.60 ± 18.10 C1: 43.29 ± 14.86 C2: 47.60 ± 14.75 C3: 54.64 ± 12.14 C4: 60.49 ± 12.44	C0: 20 men, 38 women C1: 273 men, 506 women C2: 194 men, 330 women C3: 94 men, 258 women C4: 43 men, 85 women	C0: 27.78 ± 1.48 C1: 28.99 ± 1.73 C2: 30.12 ± 2.05 C3: 30.90 ± 2.24 C4: 30.90 ± 2.01	C0: 0.17 ± 0.33 C1: 0.33 ± 0.45 C2: 0.63 ± 0.52 C3: 0.87 ± 0.66 C4: 1.39 ± 0.76	C0: −9.38 ± 3.11 C1: −11.86 ± 4.89 C2: −16.20 ± 5.42 C3: −17.04 ± 6.20 C4: −17.00 ± 5.72	High
	He et al.	2021	134 eyes 79 patients	12.32 ± 3.14	37 men, 42 women	C0: 26.48 ± 0.91 C1: 27.09 ± 0.97 C2: 28.02 ± 1.31	/	C0: −9.06 ± 1.58 C1: −9.50 ± 1.67 C2: −10.51 ± 2.76	High
	Wang et al.	2023	8330 patients	51.6 ± 13.8	5477 men, 2853 women	C0,C1: 23.67 ± 1.15 C2,C3,C4: 27.41 ± 1.12	/	/	High
India	Jonas et al.	2017	8846 eyes 4561 patients	C0,C1: 48.5 ± 12.8 C2,C3,C4: 52.8 ± 10.7	2114 men, 2447 women	C0,C1: 22.6 ± 0.8 C2,C3,C4: 29.0 ± 2.4	C0,C1: 0.11 ± 0.27 C2,C3,C4: 1.38 ± 0.90	C0,C1: −0.05 ± 1.59 C2,C3,C4: −10.6 ± 8.2	High
Japan	Hashimoto et al.	2019	2790 patients	Men: 63 ± 12 Women: 63 ± 13	1217 men, 1573 women	C2: 26.1 (25.0 to 26.6) (men), 24.8 (24.2 to 25.7) (women) C3: 27.2 (25.3 to 28.7) (men), 26.6 (24.9 to 27.8) (women) C4: 27.5 (24.0 to 31.5) (men), 27.9 (26.5 to 29.3) (women)	/	C2: −3.75 (−6.13 to −2.38) (men), −3.69 (−9.00 to −1.69) (women) C3: −6.25 (−8.94 to −2.69) (men), −6.38 (−8.75 to −5.13) (women) C4: −7.56 (−16.88 to 1.75) (men), −8.8 (women)	High
	Ueda et al.	2020	2164 patients	62.4 ± 10.9	920 men, 1244 women	C2: 25.6 ± 1.0 C3: 26.9 ± 1.5 C4: 26.0 ± 1.3	/	C2: −2.1 ± 0.9 C3: −8.0 ± 4.2 C4: −4.3 ± 3.8	High
	Fang et al.	2017	521 eyes	47.7 ± 14.2	/	C2: 29.2 ± 1.7 C3: 30.2 ± 1.9 C4: 29.7 ± 1.8	C2: 0.31 ± 0.40 C3: 0.46 ± 0.42 C4: 0.99 ± 0.43	/	High
	Du et al.	2021	3646 eyes 1877 patients	62.10 ± 12.92	520 men, 1357 women	C0, C1: 28.52 ± 1.72 (men), 28.05 ± 1.88 (women) C2: 30.22 ± 1.85 (men), 29.39 ± 2.07 (women) C3: 31.37 ± 1.96 (men), 30.73 ± 2.06 (women) C4: 30.59 ± 2.05 (men), 29.91 ± 2.06 (women)	/	/	High
	Fang et al.	2019	1487 eyes 884 patients	58.4 ± 16.3	241 men, 643 women	C0: 27.47 ± 0.89 C1: 28.51 ± 1.43 C2-PDCA: 29.33 ± 1.67 C2-MDCA: 30.55 ± 1.75 C3: 30.97 ± 2.04 C4-CNV-MA: 30.14 ± 1.91 C4-Patchy-MA: 31.73 ± 4.01	C0: −0.024 ± 0.195 C1: 0.015 ± 0.219 C2-PDCA: 0.061 ± 0.231 C2-MDCA: 0.128 ± 0.261 C3: 0.272 ± 0.403 C4-CNV-MA: 0.971 ± 0.42 C4-Patchy-MA: 0.557 ± 0.431	C0: −10.6 ± 2.8 C1: −11.4 ± 2.7 C2-PDCA: −12.9 ± 3.8 C2-MDCA: −15.2 ± 3.7 C3: −15.1 ± 4.8 C4-CNV-MA: −12.6 ± 4.4 C4-Patchy-MA: −11.1 ± 2.3	High
	Hayashi et al.	2010	806 eyes 429 patients	41.1 ± 16.7	147 men, 282 women	C1: 27.4 ± 1.2 C2: 29.5 ± 1.7 C3: 30.1 ± 2.0 C4: 30.4 ± 2.3	C1: 0.00 ± 0.15 C2: 0.23 ± 0.38 C3: 0.30 ± 0.37 C4: 1.27 ± 0.43	C1: −10.3 ± 3.1 C2: −15.2 ± 4.7 C3: −16.4 ± 5.6 C4: −17.6 ± 7.1	High
Netherlands	Haarman et al.	2021	626 patients	51.4 ± 15.1	239 men, 387 women	C2: 28.7 (28.4 to 29.1) C3: 29.6 (28.2 to 31.0) C4: 30.5 (29.5 to 31.5)	C2: 0.70 (0.64 to 0.76) C3: 0.55 (0.33 to 0.77) C4: 0.23 (0.13 to 0.34)	−9.9 ± 3.2	High
Russia	Bikbov et al.	2020	5794 patients	58.9 ± 10.7	2517 men, 3277 women	C0,C1: 23.3 ± 1.0 C2: 26.3 ± 1.8 C3: 27.3 ± 2.1 C4: 27.9 ± 2.3	/	C0,C1: 0.37 ± 2.10 C2: −6.1 ± 5.0 C3: −14.9 ± 6.5 C4: −9.8 ± 5.7	High
Singapore	Wong et al.	2019	42 eyes 29 patients	59.2 ± 11.0	11 men, 31 women (eyes)	C1: 27.35 ± 1.06 C2: 29.49 ± 1.53 C3: 30.06 ± 2.07	C1: 0.17 ± 0.10 C2: 0.27 ± 0.19 C3: 0.34 ± 0.13	C1: −8.24 ± 1.62 C2: −13.38 ± 5.72 C3: −9.88 ± 3.71	High
	Sim et al.	2020	16 patients	51.1 ± 3.0	4 men, 12 women	C1: 28.51 ± 1.73 C3,C4: 31.50 ± 1.87	C1: 0.36 ± 0.29 C3,C4: 0.39 ± 0.36	/	High
USA	Li et al.	2022	67 eyes 58 patients	C0: 55.6 ± 23.3 C2: 65.7 ± 12.8	22 men, 36 women	C0: 23.87 ± 1.04 C2: 29.67 ± 2.04	C0: 0.00 ± 0.03 C2: 0.08 ± 0.11	/	High

#### Association between axial length and the categories of myopic macular degeneration

**Fig. 2:**
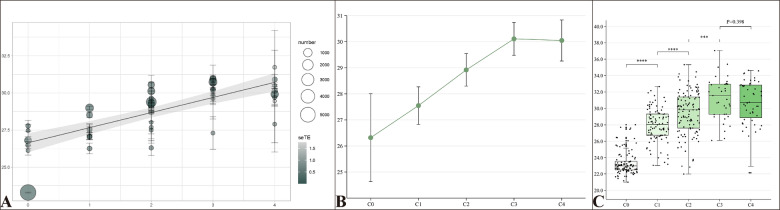
Analysis of the association between axial length (AL) and myopic macular degeneration (MMD). **A** Meta regression model for axial length (AL) and myopic macular degeneration (MMD). **B** Line graph showing the association between axial length (AL) and myopic macular degeneration (MMD). AL increased with the MMD categories change from C0 to C3, but was not significantly different between C3 and C4. **C** Box plot illustrates the distribution of axial length (AL) stratified by categories of myopic macular degeneration (MMD) based on the new data. The box plot reveals a statistically significant increase in AL as MMD severity progresses from category C0 to C3, with no significant difference observed between categories C3 and C4. Statistical significance is denoted as follows: ***P < 0.001, ****P < 0.0001.

The relationship between AL and the severity of MMD was analyzed using meta-regression, and the results were illustrated in Fig. 2A, B. The meta-regression formula, AL = 26.6424 (*P* < 0.0001) + 1.0175 category (*P* < 0.0001), indicates a significant overall increase in AL as MMD progresses from category C0 to C4, with Test of Moderators *P* < 0.0001. Furthermore, AL had a tendency to increase with greater MMD categories from C0 to C3, but this trend was not observed from C3 to C4.

### New clinical study

A total of 395 eyes of 206 participants were enrolled in the study. The mean (SD) age of the participants was 60.24 (14.53) years, the mean (SD) axial length (AL) was 27.83 (3.63) mm, and the proportion of women was 64.1%. The characteristics of the participants, stratified by categories of MMD, were shown in Table 2.

**Table 2:** Basic characteristics of participants in the new study, stratified by categories of myopic macular degeneration (MMD).

Variables	C0	C1	C2	C3	C4
No.	102	94	115	37	47
Age (year)	61.00 ± 14.12	59.17 ± 13.99	57.77 ± 15.65	57.86 ± 10.07	66.89 ± 10.04
Axial length (mm)	26.31 ± 2.50	27.96 ± 2.07	29.41 ± 2.70	31.25 ± 2.53	30.76 ± 2.62
Women [No. (%)]	68 (66.7)	59 (62.8)	71 (61.7)	22 (59.5)	36 (76.6)

#### One-way ANOVA, *t*-test, and linear mixed-effects model

The relationship between AL and the progression of MMD categories was assessed using One-Way ANOVA, t-tests, and a linear mixed-effects model, which also accounted for the correlation between binocular data from the same patients. AL increased significantly with the progression of MMD classification (*P* < 0.001). Specifically, AL showed a significant increase as MMD advanced from category C0 to C3, but no further increase was observed between categories C3 and C4 (Fig. 2C). The regression coefficient was 1.788 (95% CI, 1.542–2.035; *P* < 0.001), indicating a strong association between AL and MMD progression up to C3.

#### Ordinal logistic regression

To further explore the factors associated with the severity of MMD, ordinal logistic regression was performed. This analysis revealed that each 1 mm increase in AL was associated with a 1.9 times higher likelihood of progressing to a more severe grade of MMD (OR = 1.90; 95% CI, 1.75–2.07; *P* < 0.001). Additionally, each additional year of age increased the likelihood of advancing to a more severe MMD grade by 1.04 times (OR = 1.04; 95% CI, 1.02–1.05; *P* < 0.001), and female patients had a 1.89 times higher likelihood of progressing to more severe grades of MMD compared to males (OR = 1.89; 95% CI, 1.24–2.89; *P* < 0.01), as shown in sTable 2.

#### Multinomial logistic regression

To provide a more detailed understanding of how these factors influence specific MMD categories, multinomial logistic regression was conducted. This analysis detailed the relationship across individual MMD categories compared to the baseline category (C0). Each 1 mm increase in AL was associated with a 2.8 times higher likelihood of being classified as C1 (OR = 2.80; 95% CI, 2.19–3.59; *P* < 0.001), 3.6 times higher for C2 (OR = 3.58; 95% CI, 2.74–4.67; *P* < 0.001), 5.2 times higher for C3 (OR = 5.17; 95% CI, 3.78–7.07; *P* < 0.001), and 5.7 times higher for C4 (OR = 5.72; 95% CI, 4.17–7.84; *P* < 0.001). Similarly, age was associated with a 1.05 times higher likelihood of being classified as C3 (OR = 1.05; 95% CI, 1.00–1.09; *P* < 0.05) and a 1.14 times higher likelihood for C4 (OR = 1.14; 95% CI, 1.08–1.19; *P* < 0.001). Female patients showed a trend toward being 2.7 times more likely to be classified as C1 (OR = 2.67; 95% CI, 0.99–7.19; *P* = 0.051), although this result did not reach statistical significance. They were 3.2 times more likely to be classified as C2 (OR = 3.20; 95% CI, 1.13–9.07; *P* < 0.05), 4.0 times more likely as C3 (OR = 4.04; 95% CI, 1.16–14.09; *P* < 0.05), and 7.1 times more likely as C4 (OR = 7.12; 95% CI, 1.96–25.83; *P* < 0.01).

## Discussion

Myopia has become a public health issue worldwide due to its increasing high prevalence. The increasing prevalence of myopia and high myopia is expected to lead to a higher frequency of PM, significantly contributing to MMD, one of the most sight-threatening complications of PM, which can result in blindness or severe visual impairment [42]. AL is the strongest risk factor of MMD and serves as a reliable parameter for long-term monitoring of MMD’s onset and progression. Understanding the relationship between AL and MMD is thus of significant clinical relevance. In this study, we conducted a systematic review and meta-analysis to examine the association between AL and the severity of MMD, complemented by new data from the ophthalmic clinic at Beijing Tongren Hospital to validate our findings. Our results consistently demonstrated that AL increases with the severity of MMD.

In the meta-analysis, 20 studies were included, most of which were from Asia. According to the meta-regression, AL increased overall as MMD progresses from category C0 to C4 (*P* < 0.0001), with a tendency to increase from C0 to C3, but this trend was not observed from C3 to C4. Similarly, in the new data, we found that AL significantly increased as MMD categories progressed from C0 to C3 (*P* < 0.001), with no significant increase from C3 to C4 as determined by *t*-tests (*P* = 0.398). However, the Ordinal and Multinomial Logistic Regression analyses did not show a statistically significant decrease in the trend from C3 to C4. Therefore, while t-test results suggest a plateau in AL between C3 and C4, further research is needed to clarify this relationship. Overall, our findings are consistent with those of Yan et al. [32] and Wong et al. [43], confirming that AL generally increases with greater severity of MMD.

Additionally, each 1 mm increase in AL was associated with a 1.9 times higher likelihood of progressing to a more severe grade of MMD (OR = 1.90; 95% CI, 1.75–2.07; *P* < 0.001) in the ordinal Logistic regression model in our new data study (sTable 2). This positive correlation between AL and MMD progression is consistent with the results of the meta-analysis and other previous studies. Specifically, each 1 mm increase in AL was associated with a 2.8 times higher likelihood of being classified as C1 (OR = 2.80; 95% CI, 2.19–3.59; *P* < 0.001), 3.6 times higher for C2 (OR = 3.58; 95% CI, 2.74–4.67; *P* < 0.001), 5.2 times higher for C3 (OR = 5.17; 95% CI, 3.78–7.07; *P* < 0.001), and 5.7 times higher for C4 (OR = 5.72; 95% CI, 4.17–7.84; *P* < 0.001) (Table 3).

**Table 3:** Multinomial Logistic regression model for odds ratios (ORs) of myopic macular degeneration (MMD).

		C1	C2	C3	C4
Axial length, mm	OR (95% CI)	2.80 (2.19–3.59)	3.58 (2.74–4.67)	5.17 (3.78–7.07)	5.72 (4.17–7.84)
	P value	<0.001	<0.001	<0.001	<0.001
Age, y	OR (95% CI)	1.02 (0.99–1.04)	1.02 (0.99–1.05)	1.05 (1.00–1.09)	1.14 (1.08–1.19)
	P value	0.264	0.172	<0.05	<0.001
Gender	OR (95% CI)	2.67 (0.99–7.19)	3.20 (1.13–9.07)	4.04 (1.16–14.09)	7.12 (1.96–25.83)
	P value	0.051	<0.05	<0.05	<0.01

*CI* confidence interval, OR odds ratio.

Previous studies have consistently reported a positive correlation between AL and the prevalence of MMD [24–27, 30, 44]. Clinical studies have also demonstrated a positive correlation between AL and the severity of maculopathy [45, 46]. The underlying mechanism is thought to involve axial elongation of the globe, which leads to progressive stretching and subsequent thinning of the choroid [47, 48]. Choroidal thinning is a feature of myopia progression [49]. Numerous studies, including in animals and adults, have shown that choroids are significantly thinner in myopic eyes [50–54]. The choroid becomes thinner with axial elongation of the globe, leading to pathologic lesions and various complications in highly myopic individuals. Wong et al. demonstrated a negative correlation of choroidal thickness (CT) with MMD severity, hypothesizing that choroidal ischemia plays a critical role in the pathogenesis of MMD [43]. Chui et al. found a systematic decrease in cone photoreceptor packing density with increasing axial length, providing further evidence of retinal stretching in the myopic eye [55].

Our results of meta-analysis and new data together suggested that C4 may not be the result of progression from C3. This finding is consistent with studies by Fang et al., who reported that progression from C3 to C4 is very uncommon [3], and Zhao et al., who suggested that C3 and C4 could be considered as two different subtypes of advanced MMD [16]. In our new data, t-test analysis showed no significant difference in AL between C3 and C4 (*P* = 0.398). However, the Ordinal and Multinomial Logistic Regression models did not confirm this finding, as they did not show a statistically significant plateau or decrease in AL between C3 and C4. This discrepancy suggests that while there may be a trend toward stability in AL between these categories, further research is needed to clarify the relationship. One possible explanation for the observed discrepancy is the inherent measurement noise in AL for eyes with macular atrophy (C4), which was not mentioned in previous studies. Unlike eyes with patchy atrophy (C3), those with macular atrophy often exhibit reduced central fixation ability [56], which could increase variability in AL measurements [57, 58]. This increased “noise” may obscure subtle differences in AL between C3 and C4, potentially leading to an underestimation of true differences between these categories. This hypothesis highlights a specific instance of a broader challenge in accurately measuring AL in advanced MMD, where severe macular changes can interfere with reliable measurements. This observation underscores the need for more advanced and robust measurement methods in future studies to address these limitations.

Age is an important factor of both AL and the presence of MMD. Several population-based studies have reported that older age is independently associated with MMD [26, 29, 59–61]. Histological studies have shown that this is associated with age-related degenerative changes in the eyes, including decreased density of photoreceptor cells, ganglion cells, retinal pigment epithelium, and optic nerve fibers [62, 63]. In our new data study, each additional year of age increased the likelihood of advancing to a more severe MMD grade by 1.04 times (OR = 1.04; 95% CI, 1.02–1.05; *P* < 0.001) in the ordinal Logistic regression model (sTable 2). Moreover, age has a greater effect on C4 than C3. Age was associated with a 1.05 times higher likelihood of being classified as C3 (OR = 1.05; 95% CI, 1.00–1.09; *P* < 0.05) and a 1.14 times higher likelihood for C4 (OR = 1.14; 95% CI, 1.08–1.19; *P* < 0.001) (Table 3).

As for gender, studies had different opinions on whether it is a risk factor for MMD. Some studies found no significant association between gender and MMD [3, 25, 27, 30, 32], while others [60, 64], including our study, identified female gender as a potential risk factor for MMD progression. Our findings suggest that the influence of gender may increase with the severity of MMD, particularly in more advanced stages (C2 to C4). These varied findings across studies highlight the need for further research to explore the underlying mechanisms driving gender differences in MMD progression. The inconsistencies could be due to differences in study populations, methodologies, or other confounding factors, which warrant further investigation. Some studies on myopia suggested that women are at higher risk for myopia development, which may in turn lead to an increased risk of MMD [64]. Also, changes in the balance of sex hormones in the body may affect the physiology of the eye by sex steroid hormones (SSH) receptors in eyes [65, 66], leading to an increased risk of MMD in women. The mechanism may be related to the changes of corneal state, diopter, and ocular structure [67]. The relationship between hormonal factors and MMD needs further study. In addition, differences in genetic factors, lifestyle, and educational attainment between genders may contribute to the different risk of MMD [60].

There are several strengths of this study. To our knowledge, it is the first to directly explore the association between AL and MMD through meta-analysis, and subsequently validate these findings with reliable new clinical data. This dual approach not only provides strong evidence supporting the link between AL and MMD but also strengthens the applicability of the results within the studied population. Additionally, all studies included in the meta-analysis utilized the standardized META-PM classification system for grading fundus photographs, ensuring consistency and comparability across studies. Moreover, the quality of all included studies was assessed using clearly defined assessment tools, ensuring the quality of this meta-analysis.

This study has several limitations. First, potential biases, methodological issues, and different strategies to adjust for confounders in the original studies may have influenced the results of this meta-analysis. Second, AL measurements tend to be “noisier” in eyes with C4 compared to C3, due to the reduced ability to fixate centrally, which affects measurement accuracy. This limitation of measurement variability underscores the importance of utilizing advanced techniques for axial length measurement, particularly in eyes with severe macular atrophy. As a result, the observed differences may not be entirely attributable to pathophysiological factors, but could also reflect variations in measurement noise between these categories. Addressing these challenges in future studies could provide more definitive insights into whether C3 and C4 represent distinct subtypes of advanced MMD or are part of a continuum. Third, although our study included data from seven countries across three continents, a large proportion of participants were from China and Japan. The geographical distribution of the included studies was predominantly concentrated in East Asia, limiting the generalizability of the findings. More studies from regions such as North and South America, Africa, and Australia are needed to comprehensively explore the association between AL and MMD. Finally, the number of participants in our study was relatively small. Larger population-based studies are necessary to further validate the pattern of MMD progression and the impact of AL on it.

In conclusion, this study integrated a meta-analysis with new clinical data and employed comprehensive analytical methods. Our findings indicate that AL generally increases with the progression of MMD from category C0 to C4. However, the relationship between AL and the progression from C3 to C4 remains unclear and warrants further investigation. Future research should focus on determining whether C4 represents a distinct pathological change rather than a continuation of C3. Advanced imaging techniques, such as optical coherence tomography angiography, alongside longitudinal studies, may be essential in uncovering the specific characteristics and progression patterns of C4. The insights gained from this study may provide valuable guidance for the prevention, early diagnosis, and management of myopic macular degeneration.

## Summary

### What was known before

- High myopia has become a significant public health issue, particularly prevalent in East Asian countries. Axial length (AL)—which encompasses measurements of the anterior chamber depth, lens thickness, and vitreous chamber depth—is a critical parameter in both myopia and high myopia. Given its relevance, AL is an ideal metric for monitoring the development and progression of myopic macular degeneration (MMD). Investigating the relationship between AL and MMD holds considerable clinical significance.

### What this study adds

- The present study, consisting of a meta-analysis and a new clinical study, indicates a clear trend of increasing axial length with advancing severity of myopic macular degeneration from category 0 to category 3.

## Supplementary information

Supplementary Tables

Reproducibility Checklist

## Data Availability

The datasets generated during and/or analyzed during the current study are available from the corresponding author on reasonable request.
