# Supplementary material for: Axial length as a predictor of myopic macular degeneration: a meta-analysis and clinical study
Source: Eye (Lond). 2025 Apr 25;39(10):2073–82. doi: 10.1038/s41433-025-03782-6 (PMC12209455; doi:10.1038/s41433-025-03782-6)
Supplement: Supplementary file 1 — Supplementary Tables [file 41433_2025_3782_MOESM1_ESM.docx]

sTable1. Quality assessment of included studies in the meta-analysis.

| Study | Adequate case definition | Representativeness of the cases | Selection of controls | Definition of controls | Comparability on the most important factor | Comparability on any additional factor | Ascertainment of exposure | Same method of ascertainment for cases and controls | Non-response rate |  |
| --- | --- | --- | --- | --- | --- | --- | --- | --- | --- | --- |
| Sim et al | * | * | * | * | * | * | * | * | * | 9 |
| Hashimoto et al | * |  | * | * | * | * | * | * | * | 8 |
| Ueda et al | * | * | * | * | * | * | * | * | * | 9 |
| Zhao et al | * | * | * | * |  |  | * | * | * | 7 |
| Wang et al | * |  | * | * | * | * | * | * | * | 8 |
| Bikbov et al | * |  | * | * | * | * | * | * | * | 8 |
| Jonas et al | * |  | * | * | * | * | * | * | * | 8 |
| Yan et al | * |  | * | * | * | * | * | * | * | 8 |
| Xiao et al | * | * | * | * | * | * | * | * | * | 9 |
| Haarman et al | * |  | * | * | * | * | * | * | * | 8 |
| Fang et al | * | * | * | * | * | * | * | * | * | 9 |
| Du et al | * | * | * | * | * | * | * | * | * | 9 |
| Fang et al | * | * | * | * |  |  | * | * | * | 7 |
| Hayashi et al | * | * | * | * |  |  | * | * | * | 7 |
| He et al | * | * | * | * | * | * | * | * | * | 9 |
| Li et al | * | * | * | * | * |  | * | * | * | 8 |
| Liu et al | * | * | * | * | * |  | * | * | * | 7 |
| Wang et al | * | * | * | * |  |  | * | * | * | 7 |
| Wong et al | * | * | * | * | * |  | * | * | * | 8 |
| Zhou et al | * | * | * | * | * | * | * | * | * | 9 |

sTable 2. Ordinal Logistic regression model for odds ratios (ORs) of myopic macular degeneration (MMD).

| Parameter | OR (95% CI) | P value |
| --- | --- | --- |
| Axial length, mm | 1.90 (1.75-2.07) | <0.001 |
| Age, y | 1.04 (1.02-1.05) | <0.001 |
| Gender | 1.89 (1.24-2.89) | <0.01 |

CI=conﬁdence interval; OR=odds ratio.
